# Genetic parameters of growth traits and carcass weight of New Zealand white rabbits in a tropical dry forest area

**DOI:** 10.5455/javar.2021.h536

**Published:** 2021-09-20

**Authors:** Donicer Eduardo Montes-Vergara, Darwin Yovanny Hernndez-Herrera, Naudin Alejandro Hurtado-Lugo

**Affiliations:** 1 Faculty of Agricultural Sciences, University of Sucre, Sincelejo, Colombia; 2 University Francisco de Paula Santander Ocaña, Ocaña, Norte de Santander, Colombia

**Keywords:** Productive traits, tropical climate, genetic correlation, heritability, repeatability

## Abstract

**Objective:**

The objective of this study was to estimate the heritability (*h*^2^), repeatability (*r*), and correlations (*r_ŷ_i_y_i__*) in some traits of zootechnical interest in a population of New Zealand white rabbits of a tropical dry forest area.

**Materials and Methods:**

Three mating groups were formed, each one of 1 male and 70 females. The traits evaluated were litter size at birth (LB), born alive (BA), born dead (BD), litter weight born alive (LW), litter weight at weaning (LWW), weaning weight (WW), slaughter weight (SW), and carcass weight (CW). Weaning took place at 42 days, and the fattening phase lasted 60 ± 3 days. A descriptive statistical study was carried out on the study variables. Paternal heritability was estimated ($h_{f}^{2}$) and maternal ($h_{m}^{2}$), repeatability, rabbit index IC, and Pearson’s correlations (*r_ŷ_i_y_i__*) between traits. The descriptive statistics showed high variation for the BD traits.

**Results:**

The values of the productivity found were similar to those presented in studies around the world. *h*^2^ presented magnitudes between low and medium. $h_{f}^{2}$ ranged between 0.09 and 0.42 and between 0.11 and 0.45 for $h_{m}^{2}$. In general, the values of $h_{m}^{2}$ were higher than the values of $h_{f}^{2}$. The *r* values for the traits LB, BA, LW, LWW, and SW presented low magnitude, while it was medium for WW and CW. From the values of *r*, IC was calculated for each of the rabbits, allowing their categorization, which will be used in future selection plans. *r_ŷ_i_y_i__* among the variables ranged from −0.01 to 0.860. They were generally positive and mostly not significant (*p* > 0.05); they took a magnitude from low to moderate, except for the correlation between LB and BA.

**Conclusion:**

The production of rabbits under tropical conditions is similar to other reports. The genetic parameters evaluated were medium-to-low, indicating a robust non-additive gene and/or environmental effect.

## Introduction

Rabbit production is an economical alternative in rural and urban areas of the tropics, so its productivity is limited by the climatic conditions themselves [1]. In Colombia, rabbit production systems present little information on the genetic material available for production, which has caused producers to subjectively select replacement animals [2]. In animal genetic improvement programs, quantitative tools are applied that facilitate the selection of the best animals based on their breeding values to increase their productive and reproductive efficiency genetically [3]. In rabbits worldwide, in several productive variables, parent and offspring measurements, genetic and environmental parameters have been determined that influence the system’s productivity [4–6].

Estimation of heritability (*h*^2^), repeatability (*r*), and correlations (*r_xy_*) among the traits of economic importance at the zootechnical level is that these define the appropriate selection method; they also constitute determining factors in the selection response. For this reason, their estimation must be as precise as possible [7]. Thus, it is necessary to carry out genetic evaluation work of the reproducers, which requires characterized populations and large genetic variability. On this aspect, the success of the selection and improvement programs depends greatly [8].

There is very little research on rabbit farming in Colombia, so there is a need to carry out research projects that allow producers to implement more specialized production systems [2]. This will enable the establishment of future genetic selection programs to increase productivity [4,9]. In this sense, the objective of this work was to estimate the heritability, repeatability, and correlations in some traits of zootechnical interest in a population of New Zealand white (NZW) rabbits in a tropical dry forest area.

## Materials and Methods

### Study location

The present study was carried out in the experimental farm “Los Pericos” of the Universidad de Sucre, located in the municipality of Sampués, Colombia (9°15ʹ North and 71°22ʹ54ʺ West), at an altitude of 202 masl, an area belonging to the tropical dry forest [10] or alternative-hydrogen tropical zonobiome [11]. The average temperature is 27°C, with a maximum of 32°C and a minimum of 21°C. The average precipitation, relative humidity, and wind speed values are 1,200 mm, 75%, and 10.2 km/h, respectively.

### Experimental animals and procedures

The current study was approved by the Committee of Animal Care and Welfare, Sucre University, Colombia (approval number 13-2019). The animals were raised on a closed farm with natural ventilation. The males were housed in a single cylindrical cage 70 cm in diameter and 1 m high. The females were housed individually in galvanized metal cages with dimensions 50 × 90 × 35 cm, duly equipped with nests, feeders, and automatic drinkers, obeying a flatdeck system. The feeding of all rabbits was based on a commercial diet with 17% crude protein, 2.5% fat, 12% ash, 13% moisture, 15% fiber, and 3,200 kcal/kg of metabolizable energy with water *ad libitum*.

The population of rabbits used consisted of animals of the NZW breed, obtained under a planned reproductive scheme during 2019 and 2020. Three males and 210 females were used. Three mating groups were formed containing 70 females, and 1 male was presented to the corresponding male in a nested design. The breeding stallion was assumed as the father of the entire litter. Pregnancy was determined by palpation 10 days after mating. Non-pregnant females were covered again 12 h later, with the same male. The kits were identified, following racial patterns and groups. Weaning was carried out at 42 days, then grouped by sex and taken to cages for the fattening phase of 60 ± 3 days. The animals fasted for 12 h before sacrifice. After fasting, the rabbits were weighed (SW) on a precision analytical balance (± 1 gm). The animals were stunned by electronarcosis and slaughtered by slitting the throat; the skin, head, legs were removed, and viscera [2]. The resulting carcasses were weighed.

### Analysis of data

The productive parameters evaluated were litter size at birth (LB), born alive (BA), born dead (BD), litter weight born alive (LW), litter weight at weaning (LWW), weaning weight (WW), slaughter weight (SW), and carcass weight (CW). A descriptive analysis was carried out, in which the means, standard error, and coefficients of variation (CV) were estimated for the variables LB, BA, BD, LW, LWW, WW, SW, and CW. In addition, the assumptions of normality, independence, and homoscedasticity were validated using different procedures of the statistical program SAS (2021), where no significant deviations were found from the assumptions (*p* > 0.05).

Analysis of variance was carried out for all variables, using the ProcGLM procedure of SAS (2021), to measure the significance of the fixed effects considered on the respective parameters studied, using the linear additive model as follows:

$$Y_{\mathrm{ijk}}=\mu +A_{i}+B_{\mathrm{ij}}+{ɛ}_{\mathrm{ijk}}$$

where *Y_ijk_* is the observed value of the variables studied (LB, BA, BD, LW, LWW, WW, SW, and CW), ∞ is the effect of the general mean of the traits studied, *A_i_* corresponds to the random effect of the *i*-th parent (1:3), *B_ij_* is the random effect of the *j*-th mother paired with the *i*-th father (1:21), and *ε_ijk_* is the random error.

Using the SAS MIXED procedure, heritability (*h*^2^), paternal and maternal, was estimated from the variance component of the father and mother, using the following formulas [7]:

$$h_{f}^{2}=\frac{4\sigma_{f}^{2}}{\sigma_{f}^{2}+\sigma_{m}^{2}+\sigma_{w}^{2}};\;\;h_{m}^{2}=\frac{4\sigma_{m}^{2}}{\sigma_{f}^{2}+\sigma_{m}^{2}+\sigma_{w}^{2}}$$

where $h_{f}^{2}$ and $h_{m}^{2}$ are the paternal and maternal heritabilities, respectively; $\sigma_{f}^{2}$ is the variance component of the parent; $\sigma_{m}^{2}$ is the variance component of the mother; and $\sigma_{w}^{2}$ is the variance component within the progeny. The standard error of *h*^2^ was calculated with the following formula:

$$\mathrm{SE}(h^{2})=4\sqrt{\frac{2(N-1\mathrm{)(}1-t{)}^{2}[1+(k-1)t{]}^{2}}{k^{2}(N-S\mathrm{)(}S-1)}}$$

where *t* is hf214 or hm214, depending on the case; *k* is the mean number of progenies per breeder; and *N* and *S* are the total number of records and the total number of reproducers (father or mother depending on the case), respectively.

Repeatability and its standard error were estimated from the variance components between and within rabbits, obtained by the SAS MIXED procedure, and thus:

$$r=\frac{\sigma_{m}^{2}}{\sigma_{m}^{2}+\sigma_{e}^{2}};\;\;\mathrm{SE}(r)=\sqrt{\frac{2(1-r{)}^{2}[1+(k-1)r{]}^{2}}{k(k-1\mathrm{)(}N-1)}}$$

where *r* is the repeatability, SE(*r*) is the standard error of repeatability, $\sigma_{m}^{2}$ is the variance component of the mother, $\sigma_{e}^{2}$ is the variance component within mothers (error), and *N* and *S* are the numbers of rabbits and measurements per individual, respectively [7]. The repeatability values were used to estimate the index of rabbit female (IC) of each of the traits studied, in order to maintain the mothers with the highest genotypic value, due to their productive capacity in future births by applying the following equation [7]:

$$\mathrm{IC}=\overset{─}{X_{p}}+\frac{\mathrm{nr}}{1+(n-1)r}({\overset{─}{X}}_{i}-{\overset{─}{X}}_{p})$$

where IC is the doe index, ${\overset{─}{X}}_{p}$ is the mean of the group studied for a particular trait, ${\overset{─}{X}}_{i}$ is the mean of the rabbit for a particular trait, *n* is the number of records for each rabbit, and *r* is the repeatability value. From the ICs for each variable, the rabbits were classified from highest to lowest to establish which animals could be destined to produce the future breeders of the farm.

Finally, the correlation coefficient between the different traits studied was estimated through Pearson’s correlation, using the following formula [12]:

$$r_{{ŷ}_{i}y_{i}}=\frac{\mathrm{covarianza}({ŷ}_{i}y_{i})}{\sqrt{\sigma_{{ŷ}_{i}}^{2}\sigma_{y_{i}}^{2}}}$$

where *r_ŷ_i_y_i__* is the correlation coefficient and *ŷ_i_* y *y_i_* are the predicted and observed phenotypic values, respectively.

## Results and Discussion

Table 1 shows the descriptive statistics for the traits studied. The traits, in general terms, presented CV lower than 20%, similar to that presented by Okoro et al. [13] and El-Atrouny et al. [9]. However, in variable BD, the CV value exceeded 70%. These indices allow identifying the traits of most significant interest to be used in genetic improvement programs in rabbits since they quantify the variability between individuals. However, it is necessary to determine what proportion of this variation is due to genetic effects.

The mean LB value obtained here was higher than that presented by Gambo et al. [4] and Adeolu et al. [8], but lower than the reports by Badawy et al. [14] and Pycha et al. [15]. The differences found between the different reports for BL may be due to the disparity in the conception rate and the maternal effect, which is generally determined by the number of eggs matured, fertilized, and implanted by the doe [8]. In this sense, it has been determined that the size of the litter has a significant effect on the future performance of the litter [16], so the lower the number of kits per mother, the higher the birth weight, body weight gain, and growth rate [4].

The result of BA and DB were similar to those reported in the literature for the same breed by Pycha et al. [15], although Pollesel et al. [16] have reported values higher than those found in this study. It has been reported that animals with higher birth weights have higher body weight at the first service and during their entire productive cycle [17]. In addition, it correlated low birth weights with lower survival, although many other environmental factors exacerbate this effect. Agea et al. [18] reported higher weights for the LW variable, and Fadare and Fatoba [19] presented lower weights than those found in this work. 

From the results presented in Table 1, it is inferred that the average weight of the kits at birth was 49.75 gm. Agea et al. [18] point out that kits weighing less than 50 gm require temperature control in the nest and greater consumption of colostrum, in favor of adequate nutrition, which is summarized in greater maternal ability. Thus, the time of calving, milk intake, and the lactation parity status of the female affect the probability of survival and other factors.

The LWW is determined by the number of kits born and the mother’s milk capacity since she maintains the homogeneous weight in the litter, reduces competition in the udder, and increases its viability [9]. The LWW in this study was similar to that of Agea et al. [18] and lower than the finding of El-Deghadi [3]. Then, the weight of the kits at weaning (WW) depends on the size of the litter, the weight being lower when the litter size is high [20]; from Table 1, it is inferred that the average size of the litter at weaning was 5.68 kits, with a WW of 423.7 ± 14.5 gm. This weight was similar to that presented by Agea et al. [18] and Khan et al. [5], but lower than those found by Amao [21] and Fang et al. [22]. The differences observed in the literature can be attributed to the mother’s milk yield, maternity capacity, and the management during rearing in each farm.

**Table 1.** Descriptive statistics of litter traits in a population of NZW rabbits in a tropical dry forest area.

Litter traits	No. of data	Mean (± SE)	CV%
LB (*n*)	210	6.9 ± 0.94	18.4
BA (*n*)	1,330	6.33 ± 0.79	12.5
BD (*n*)	140	0.58 ± 0.21	72.0
LW (gm)	210	314.7 ± 13.2	15.1
LWW (gm)	210	2,407.9 ± 22.7	13.9
WW (gm)	1,280	423.7 ± 14.5	11.3
SW (gm)	1,280	2,347.3 ± 32.3	10.8
CW (gm)	1,280	1,281.6 ± 19.6	13.1

SE: standard error. CV: coefficient of variation.

The SW was similar to previous reports [2,23], higher than those presented by Sánchez et al. [24], but lower than those presented by Matics et al. [25]. The variation between the stated averages is due to differences in breeds, temperature, season, slaughter age, and food quality. It has also been shown that ambient temperature over thermoneutrality values reduces feed consumption and, consequently, decreases growth speed [25]. The CW for the studied rabbits was found within the range of 1,250–1,480 gm/animal, stated by several authors [2,23]. However, authors such as Matics et al. [25], Rasskazova et al. [26], and Ayyat et al. [27] found higher CW in a range oscillating between 1,700 and 1,890 gm/animal. Rotımı et al. [28] suggested that the differences in weights before slaughter and in the hot carcass could be attributed to age, sex, breed, feeding conditions, and management to which rabbits are subjected on the farm to the slaughter method. The above is a possible explanation for the differences found in the literature reports.

Table 2 shows the values of *h*^2^ and *r* for the LB, BA, BD, LW, LWW, WW, SW, and CW traits. The calculated values of *h*^2^ had a magnitude between low to medium, oscillating between 0.09 and 0.42 when it was estimated from the paternal component ($h_{f}^{2}$ ) and between 0.11 and 0.45 when estimated from the maternal component ($h_{m}^{2}$ ). In general, the values of $h_{m}^{2}$ were higher than the values of $h_{f}^{2}$. In this sense, Sorhue et al. [29] observed similar behavior and suggest the improvement of these, using progeny tests or the analysis of the siblings, since *h*^2^ depends on the variance of the population. Estimates dropped from *h*^2^ allow inferring that the variation due to environmental factors or the non-additive effects of genes (dominance, over-dominance, and epistasis) is probably higher than their additive effects. On the contrary, estimates of *h*^2^ of high magnitudes reveal a strong additive genetic effect [6].

The estimate of *h*^2^ obtained for LB in the present work is similar to those reported in the literature [30]. However, Adeolu et al. [8] found higher values, and El-Atrouny et al. [9] and Kosba et al. [31] reported lower values. *h*^2^ of BA was considered to be of low magnitude. Heritability estimates for this range vary considerably in the literature, reporting from low to moderate values [6,32,33]. About the BD traits, *h*^2^ estimated was higher than those reported previously [6,14], using different estimation models. The values of *h*^2^ estimated for LW and LWW were considered means, with a greater magnitude than that reported by Peiró et al. [34] and Gharib et al. [35] and with a lower value than that presented by Fayeye and Ayorinde [36]. *h*^2^ values estimated for WW and SW were similar to previous reports [9,37]. On the other hand, estimates reported by Peiró et al. [34], Badawy et al. [14], and Ezzeroug et al. [32] had lower magnitudes. On the contrary, Moustafa et al. [38] found higher values of 0.47 and 0.33 for WW and SW, respectively. The found value of *h*^2^ for CW is considered low, and a similar value was reported previously [39]. Superior reports have been mentioned by Hervé et al. [40].

**Table 2.** Estimates of heritability, repeatability, and standard errors for litter traits of NZW rabbits in a tropical dry forest area.

Litter traits	$h_{f}^{2}$ ± SE	$h_{m}^{2}$ ± SE	*r* ± SE
LB (*n*)	0.09 ± 0.15	0.13 ± 0.16	0.12 ± 0.09
BA (*n*)	0.11 ± 0.23	0.12 ± 0.11	0.15 ± 0.10
BD (*n*)	0.09 ± 0.22	0.25 ± 0.12	0
LW (gm)	0.35 ± 0.22	0.42 ± 0.31	0.23 ± 0.32
LWW (gm)	0.42 ± 0.18	0.45 ± 0.32	0.18 ± 0.09
WW (gm)	0.26 ± 0.12	0.29 ± 0.21	0.38 ± 0.14
SW (gm)	0.10 ± 0.16	0.16 ± 0.17	0.21 ± 0.11
CW (gm)	0.09 ± 0.13	0.11 ± 0.15	0.42 ± 0.12

$h_{f}^{2}$: paternal heritability; $h_{m}^{2}$: maternal heritability; *r*: repeatability; SE: standard error.

The great variation found in the values of *h*^2^, which are presented in the literature and what is presented here, justifies the effect of the non-additive gene action and the environment on the evaluated traits. In this regard, Garcia et al. [33] also indicate that the size of the evaluated population, the different managements given in each farm, the productive capacity of each mother, the bodyweight of the adults, the growth rate, the maturity at the time of slaughter, the ages at weaning and slaughter, and the prediction models used for the calculation are factors that explain these differences.

A measure of the similarity of the performance of individuals in successive litters throughout the yield cycle is repeatability. It depends on both genotypes and environmental factors [15]. Estimates of *r* for the evaluated traits ranged between 0.12 and 0.42 (Table 2). *r* for traits LB, BA, LW, LWS, and SW presented a low magnitude, indicating a strong influence of environmental factors. Similar results for various litter-related traits were presented by Pycha et al. [15], Behiry et al. [30], and Karim et al. [41]. For the WW and CW traits, the values obtained from *r* tend to be moderate; similar reports were presented previously [3,9].

From *r* values for traits LB, BA, LW, LWW, WW, SW, and CW, the IC was calculated for each of the rabbits under study (Table 3). This value allowed to classify and identify the best rabbits according to the traits under investigation. The IC score indicates that a female rabbit in the following calving period can repeat its productivity, and the average production of the evaluated variable may be higher than that of its population. In this sense, superior rabbits will have a higher IC score than their reference population [9]. The CI estimates are helpful for a programmed selection, which helps predict the correlative response to the selection. This ultimately helps to choose the breeding system to follow for future improvement and increasing genetic gain. This index enables making changes in the population dynamics due to selection, pressure, and slaughter. It can be used as an alternative for the choice of breeders on farms where genetic values are not available [42].

**Table 3.** Classification of rabbits according to IC in relation to the variables LB, BA, LW, LWW, WW, SW, and CW.

Order	LB		BA		LW		LWW		WW		SW		CW	
	Doe	IC	Doe	IC	Doe	IC	Doe	IC	Doe	IC	Doe	IC	Doe	IC
1	C6	7.12	1A	6.6	6A	320.98	2A	2,925.5	2B	478.2	C4	2,442.1	2B	1,309.3
2	C5	7.12	4A	6.4	C1	319.37	6A	2,925.2	C5	477.8	C5	2,397.4	C5	1,307.2
3	C2	7.12	5A	6.4	4B	318.68	7A	2,924.6	3A	473.3	C7	2,389.6	7B	1,298.6
4	C1	7.12	3B	6.4	C2	317.07	1A	2,923.9	7A	470.2	C2	2,379.1	7A	1,297.4
5	5A	7.12	4B	6.4	C3	317.07	4B	2,922.5	3B	470.2	6A	2,370.1	4A	1,297.0
6	4B	7.12	C1	6.4	7B	316.38	4A	2,921.4	4A	469.5	C6	2,361.1	1B	1,297.0
7	1A	7.12	C2	6.4	6B	315.92	C7	2,921.4	C6	468.7	2A	2,358.1	6B	1,297.0
8	C7	7.0	C5	6.4	C4	315.92	1B	2,920.1	4B	467.9	4A	2,358.1	C7	1,292.9
9	C4	7.0	C6	6.4	3B	314.77	6B	2,918.3	6B	466.4	1A	2,353.9	3A	1,288.8
10	C3	7.0	2A	6.3	C5	314.77	3B	2,914.7	1B	465.7	3B	2,353.9	6A	1,288.8
11	7B	7.0	3A	6.3	1A	314.54	7B	2,909.3	C2	462.6	C3	2,353.9	5A	1,284.7
12	7A	7.0	7A	6.3	2B	314.08	C3	2,907.5	5A	461.9	C1	2,349.3	4B	1,284.7
13	4A	7.0	2B	6.3	3A	313.62	3A	2,905.7	5B	461.5	7A	2,337.1	1A	1,280.6
14	3B	7.0	5B	6.3	5A	313.62	5A	2,900.3	6A	460.3	5B	2,337.1	5B	1,280.6
15	2B	7.0	7B	6.3	7A	313.62	2B	2,900.3	C4	460.3	3A	2,322.0	C6	1,280.6
16	2A	7.0	C3	6.3	5B	313.62	5B	2,891.3	C7	459.6	5A	2,320.3	C3	1,270.3
17	5B	6.88	C4	6.3	C7	313.62	C6	2,890.4	7B	458.8	7B	2,320.3	C2	1,258.0
18	3A	6.88	C7	6.3	C6	312.47	C2	2,889.9	1A	458.1	2B	2,316.1	C1	1,256.0
19	1B	6.88	6A	6.1	4A	310.17	C4	2,884.1	C3	447.0	6B	2,298.3	2A	1,247.8
20	6B	6.76	1B	6.1	2A	309.48	C1	2,880.4	2A	446.7	4B	2,290.5	3B	1,243.7
21	6A	6.76	6B	6.1	1B	309,02	C5	2,873.2	C1	443.6	1B	2,276.2	C4	1,243.7

IC: rabbit index; LB: litter size at birth; BA: born alive; LW: litter weight born alive; LWW: litter size at weaning; WW: weaning weight; SW: slaughter weight; CW: carcass weight.

Table 4 shows the estimation of the correlations between the variables under study, taking father and mother as a source. The estimates ranged from −0.01 to 0.860; they were generally positive and mostly not significant (*p* > 0.05); they took a low-to-moderate magnitude, except for LB and BA, which presented high values. The variables LB and BA, LB and BD, and WW and CW were positive, with statistically significant results (*p* < 0.05) for both sources (father and mother). The correlation estimates between LB and BA and LB and BD were within the ranges reported in the literature [9,33,43]. However, it must be considered that in large litters, mean LW decreased and that LW is related to various patterns associated with maternal ability and productive and reproductive traits [16,17]. The value of the correlation between WW and CW can be considered relevant; similar results were reported by Badawy et al. [14] and Peiró et al. [34], who consider that values greater than 0.30 are considered relevant since it represents less than 10% of the variance of a characteristic explained by the other, as well as the probability of similarity. The relationship between the SW and CW variables was statistically significant (*p* < 0.05), similar to that found previously [2,28,44,45].

## Conclusion

The reproductive and productive performance of NZW rabbits reared in tropical conditions did not show marked differences with reports worldwide. The repeatability estimates for all the studied traits maintained a low to moderate magnitude, evidencing the need to increase the number of observations to improve the precision in the estimation. Low magnitude heritability indicates that they can be enhanced by progeny registration or sibling testing. The magnitudes of repeatability were low to moderate. Its applicability allowed the rabbits to be scored according to the IC and establish a classification used to select the best animals. The selection in characteristics with positive genetic correlation will lead to a progress in genetics on the other traits. It is the case between SW and CW which implies that SW could be used as a selection criterion for the characteristics of the carcass. On the contrary, the negative correlations indicate that selecting one of the characteristics could decrease the other.

**Table 4.** Estimate of phenotypic correlation for litter traits of NZW rabbits in a tropical dry forest area.

Litter traits	LB	BA	BD	LW	LSD	WW	SW	CW
LB	1.00							
						
BA	0.86*	1.00						
	0.79*							
BD	0.55*	0.04	1.00					
	0.49*	0.06						
LW	0.01	0.02	−0.02	1.00				
	0.03	0.03	−0.04					
LWW	−0.39	−0.28	−0.31	−0.2	1.00			
	−0.45	−0.32	−0.43	−0.16				
WW	−0.08	0.02	−0.18	−0.22	−0.06	1.00		
	−0.06	0.04	−0.19	−0.18	−0.03			
SW	0.25	0.24	0.09	0.15	−0.4	−0.16	1.00	
	0.19	0.31	0.06	0.12	−0.36	−0.11		
CW	−0.22	0.24	0.04	−0.14	0.18	0.56*	0.44*	1.00
	−0.19	0.28	0.02	−0.12	0.21	0.51*	0.42*	

LB: litter size at birth; BA: born alive; LW: litter weight born alive; LWW: litter size at weaning; WW: weaning weight; SW: slaughter weight; CW: carcass weight. First value: sire component, second value, dam component.

* Correlations are significant (*p* < 0.05).

## List of Abbreviations

LB: litter size at birth; BA: born alive; LW: litter weight born alive; LWW: litter size at weaning; WW: weaning weight; SW: slaughter weight; CW: carcass weight; $h_{f}^{2}$ : paternal heritability; $h_{m}^{2}$ : maternal heritability; *r*: repeatability.
